# Supplementary material for: Gamete binning: chromosome-level and haplotype-resolved genome assembly enabled by high-throughput single-cell sequencing of gamete genomes
Source: Genome Biol. 2020 Dec 29;21:306. doi: 10.1186/s13059-020-02235-5 (PMC7771071; doi:10.1186/s13059-020-02235-5)
Supplement: Supplementary file 3 — Additional file 3. Review history. [file 13059_2020_2235_MOESM3_ESM.docx]

**1^st^ round**

**Reviewer 1**

Schneeberger and colleagues present a new method for generating haplotype-resolved genome assemblies that uses gamete genomes, rather than parental genomes, for phasing information. By sequencing many recombined, single-cell gamete genomes, they are also able to construct a genetic map that can be used for scaffolding the resulting haplotypes. It is a sound and clever idea, but the practicality is questionable due to its reliance on single-cell sequencing and lack of comparisons to competing techniques other than trio binning. The proposed approach does have the added benefit of studying meiotic recombination in the gamete genomes themselves, which is a nice addition to the paper.

1) One major omission is any reference to competing phasing approaches other than trio binning.

Strand-Seq, which is a single-cell technique that requires neither parents nor gametes, can be used to cluster long sequencing reads by chromosome, phase haplotypes, and scaffold using genetic map techniques (similar to the approach proposed here). E.g.
Falconer et al. 2012 https://doi.org/10.1038/nmeth.2206
Hills et al. 2018 https://doi.org/10.1101/271510
Chaisson et al. 2019 https://doi.org/10.1038/s41467-018-08148-z

Hi-C has also been used to directly assemble and phase chromosomes, and it is a much easier datatype to generate than single-cell Strand-Seq or gamete sequencing. E.g.
Selvaraj et al. 2013 https://doi.org/10.1038/nbt.2728
Garg et al. 2019 https://doi.org/10.1101/810341

At the very least, some discussion of the pros/cons of these existing approaches vs. the proposed approach is needed.


2) A second major omission is any structural validation of the final assemblies. How does the accuracy of the genetic map scaffolding compare to the competing scaffolding approaches of Hi-C, Bionano, etc.? The synteny comparisons to a related species are not very convincing. I would expect the genetic map would be prone to local-style mis-assemblies (inversions and local rearrangements), which synteny analyses will not reveal. A more rigorous structural validation would include some complementary data such as long nanopore reads, Hi-C maps, or optical maps.


3) Without solid validation of the structural quality of the scaffolds, it is very difficult to interpret something like Figure 4. What is the evidence that these inversions and rearrangements are biological, and not just artifacts of the assembly process?


4) What is the accuracy of the assembly consensus, in terms of QV? Is there any evidence of duplicated sequence between the haplotypes (e.g. a single allele artificially duplicated in both haplotypes)? KAT plots can also be handy for demonstrating the quality of a diploid assembly. You might consider including one (Mapleson et al. 2016 https://doi.org/10.1093/bioinformatics/btw663)


5) Please also include the contig stats in Table 1, in addition to scaffold stats. Despite the high sequencing coverage, the contigs seem relatively fragmented for a long read assembly (~500 kbp N50). What is causing this?

**Reviewer 2**

Dear Colleagues,

I read with interest your manuscript on a new strategy to resolve haplotypes through high-throughput single-cell sequencing of gamete genomes. This is particularly important for highly variably species, such as plants. As you point out, resolving chromosome-length haplotypes is still very complex (even on basic species such as human), and numerous strategies, ranging from genetics to long read assays have already been attempted. This is the first time to my knowledge that someone uses gametes to do this, and the approach is very elegant.

The text itself is quite clear.

One question that occurred to me upon reading was how competitive this approach is compared to existing alternatives. E.g. why not simply use sufficiently long reads to resolve the haplotypes? You answer this question indirectly in studying the distance between markers, but this bears discussion. However, what about other techniques such as Hi-C etc?

Excellent work,

Daniel Zerbino

**Authors’ response**

Reviewer 1:

General comment: Schneeberger and colleagues present a new method for generating haplotype-resolved genome assemblies that uses gamete genomes, rather than parental genomes, for phasing information. By sequencing many recombined, single-cell gamete genomes, they are also able to construct a genetic map that can be used for scaffolding the resulting haplotypes. It is a sound and clever idea, but the practicality is questionable due to its reliance on single-cell sequencing and lack of comparisons to competing techniques other than trio binning.

The proposed approach does have the added benefit of studying meiotic recombination in the gamete genomes themselves, which is a nice addition to the paper.

*Thank you very much for the supportive evaluation.*

*However, we respectfully disagree with assessment that the practicality of our method is questionable. With the advent of single cell sequencing technologies like the 10X Genomics Controller, high-throughput single cell sequencing now becomes widely accessible. While it still might not be straightforward to isolate individual nuclei, we expect that the development of the single-cell technologies has not reached its end yet, and more easy-to-handle technologies will come to market soon – which finally will make the application of gamete binning as easy as the application of Hi-C (which itself used to be a delicate technology that was only performed in dedicated research groups).*

*Having said this, we do agree with the reviewer that the comparison of gamete binning to competing techniques is interesting. We have therefore generated Hi-C data from the apricot tree and performed a comparison of gamete binning with Hi-C-based assembly, which is now described in the main manuscript.*

Comment 1: 1) One major omission is any reference to competing phasing approaches other than trio binning.

Strand-Seq, which is a single-cell technique that requires neither parents nor gametes, can be used to cluster long sequencing reads by chromosome, phase haplotypes, and scaffold using genetic map techniques (similar to the approach proposed here). E.g.

Falconer et al. 2012 <https://doi.org/10.1038/nmeth.2206>

Hills et al. 2018 <https://doi.org/10.1101/271510>

Chaisson et al. 2019 <https://doi.org/10.1038/s41467-018-08148-z>

Hi-C has also been used to directly assemble and phase chromosomes, and it is a much easier datatype to generate than single-cell Strand-Seq or gamete sequencing. E.g.

Selvaraj et al. 2013 <https://doi.org/10.1038/nbt.2728>

Garg et al. 2019 <https://doi.org/10.1101/810341>

At the very least, some discussion of the pros/cons of these existing approaches vs. the proposed approach is needed.

*We have added detailed discussions about the pros and cons of these approaches including the publications described above to the Introduction of our manuscript.*

*In addition, we deeply sequenced two Hi-C libraries and performed an actual comparison of Hi-C vs. gamete binning based genome assembly. After aligning the reads against an initial haplotype-resolved assembly from Canu*^1^*, we used the ALLHiC pipeline*^2^ *to phase and scaffold the diploid genome into 16 groups (note that we also tried SALSA2 for Hi-C based genome phasing and scaffolding, which however did not generate a chromosome-level assembly but instead resulted in over 2,700 scaffolds).*

*First, we compared a Hi-C contact map based on the gamete binning assembly (rFig. 1a) against a contact map based on the ALLHiC assembly (rFig. 1b). The contact signals surrounding the main diagonal line were much weaker in the ALLHiC assembly including many contacts between genomic regions that were distantly scaffolded, which indicates that some of the contigs were likely to be scaffolded in the wrong order. Moreover, there were hardly any contacts captured between homologous chromosomes in the ALLHiC assembly, which indicates that the assembly was falsely combining sequences from different haplotypes.*

*Secondly, in almost all haploid chromosome sequences of the ALLHiC assembly we found significant amounts of parent-specific k-mers from both of the parental genomes indicating severe errors in haplotype phasing (rFig. 1c). Performing a whole-genome alignment of the Hi-C assembly against a genetic map derived assembly (i.e. the gamete binning assembly), we found that many of the ALLHiC based scaffolds include regions from different linkage groups indicating severe scaffolding errors in addition to the phasing errors (rFig. 1d).*

*Taken together, somewhat unexpected and despite the broad usage of Hi-C for genome assembly, Hi-C based genome assemblies faced great challenges in phasing and scaffolding, while gamete binning was highly accurate in genome phasing (and similar in performance to trio binning). The weak performance of the Hi-C assembly are likely to result from the actual contacts between homologous chromosomes that mislead phasing and scaffolding (rFig. 1a).*

Comment 2: 2) A second major omission is any structural validation of the final assemblies. How does the accuracy of the genetic map scaffolding compare to the competing scaffolding approaches of Hi-C, Bionano, etc.? The synteny comparisons to a related species are not very convincing. I would expect the genetic map would be prone to local-style mis-assemblies (inversions and local rearrangements), which synteny analyses will not reveal. A more rigorous structural validation would include some complementary data such as long nanopore reads, Hi-C maps, or optical maps.

Comment 3: 3) Without solid validation of the structural quality of the scaffolds, it is very difficult to interpret something like Figure 4. What is the evidence that these inversions and rearrangements are biological, and not just artifacts of the assembly process?

*We have used the new Hi-C data also to validate all large-scale rearrangements (>500kb) that we found between the two haplotype assemblies (examples shown in rFig. 2) and could confirm each single one of them. In general, the contiguity of the contact map signals at the main diagonal of the map demonstrates the high quality of the structure of the gamete binning assemblies (rFig. 1a) and thus allow the comparison of SV vs. meiotic recombination in this genome.*

Comment 4: 4) What is the accuracy of the assembly consensus, in terms of QV? Is there any evidence of duplicated sequence between the haplotypes (e.g. a single allele artificially duplicated in both haplotypes)? KAT plots can also be handy for demonstrating the quality of a diploid assembly. You might consider including one (Mapleson et al. 2016).

*We have performed k-mer based assembly evaluations using KAT*^3^ *and Merqury*^4^ *(rFig.3 and 4). The Merqury analysis showed that our assemblies have a QV of over 36, which is highly comparable to reported assemblies*^4^*. We hardly observed any signal for duplicated regions. Details and related discussions are now included in the main text.*

Comment 5: 5) Please also include the contig stats in Table 1, in addition to scaffold stats. Despite the high sequencing coverage, the contigs seem relatively fragmented for a long read assembly (~500 kbp N50). What is causing this?

*Thank you for the suggestion, and we have now added contig stats to Table 1. The “fragmented” assemblies are most likely a result of the (error-prone) PacBio reads from the Sequel I platform with a mean length of “only” 8 kb. However, despite this contig fragmentation, we could still scaffold the vast majority of these contigs into chromosomes. We believe that with the broad availability of highly accurate long reads such as the PacBio HiFi reads, our method would become be even more powerful.*

Reviewer 2:

One question that occurred to me upon reading was how competitive this approach is compared to existing alternatives. E.g. why not simply use sufficiently long reads to resolve the haplotypes? You answer this question indirectly in studying the distance between markers, but this bears discussion. However, what about other techniques such as Hi-C etc?

*Despite of some first successful reports, long reads are generally still not sufficient to accurately resolve haplotypes and assemble full chromosomes of complex genomes. While we have no doubt that this will be the future, long tracks of homozygous regions and/or repeats between homologous chromosomes can still be challenging to correctly un-tangle the haplotypes from assembly graphs.*

*In the new version of the manuscript, we included newly generated Hi-C data, and use it to compare gamete binning with Hi-C-based phasing and scaffolding (please also see our second response to reviewer 1 for details). Unexpectedly, the Hi-C based methods such as ALLHiC or SALSA2 could not properly phase the haplotypes and generated many more mis-joins and phase errors as compared to gamete binning (rFig. 1; rFig. 2).*

References

1.	Koren, S. *et al.* Canu: scalable and accurate long-read assembly via adaptive k-mer weighting and repeat separation. *Genome Res.* **27**, 722–736 (2017).

2.	Zhang, X., Zhang, S., Zhao, Q., Ming, R. & Tang, H. Assembly of allele-aware, chromosomal-scale autopolyploid genomes based on Hi-C data. *Nat. Plants* **5**, 833–845 (2019).

3.	Mapleson, D., Garcia Accinelli, G., Kettleborough, G., Wright, J. & Clavijo, B. J. KAT: a K-mer analysis toolkit to quality control NGS datasets and genome assemblies. doi:10.1093/bioinformatics/btw663

4.	Rhie, A., Walenz, B., Koren, S. & Phillippy, A. Merqury: reference-free quality, completeness, and phasing assessment for genome assemblies. 1–41 (2020). doi:10.1101/2020.03.15.992941

**rFig 1 (new Figure 4 in the main text). Comparison of Hi-C vs. gamete binning based assemblies. a.** Hi-C contact map based on the gamete binning assembly (including both haplotypes Currot (CU) and Orange Red (OR)). **b.** Hi-C contact map based on the Hi-C based assembly. Contact signals along the main diagonal were much weaker as compared to the gamete binning assembly. Contact signal between two different haplotypes could not be identified. **c.** Haplotype validation of each linkage group (LG-1-8) of the Hi-C assembly using parent-specific k-mers. Almost all haplotype-specific assemblies included k-mers specific to both of the parental alleles indicating severe errors in the phasing. **d.** Alignment of the Hi-C based assembly to genetic map derived assembly (i.e. gamete binning derived assembly) revealed mis-joining or splitting of linkage groups within the Hi-C assembly.

**rFig. 2 (new Fig. 3e). Hi-C contact map** based on the gamete binning assemblies of the two haplotypes of chromosome 1 (Currot (CU) and Orange Red (OR)) at a resolution of 300 kb. The contact signal showed a high contiguity within the haplotypes (main diagonal line) and confirmed two large inversions (v_11_ and v_12_) which we observed in the assembled sequence of the two haplotypes. (See Supplementary figures 9-15 for chromosomes 2-8).

rFig. 3 (new Supplementary Figure 7). *k*-mer comparison plot for genome assembly evaluation by KAT3^3^. Parental (‘Rojo Pasión’) read *k*-mer frequency versus Orange Red haplotype (**a**), Currot haplotype (**b**) and Rojo Pasión (Orange Red and Currot haplotypes merged) (**c**) copy number stacked histograms. Read content in black is absent from the assembly, red occurs once, purple twice, etc. *k*-mer spectra show an error distribution under 45x, heterozygous content around 85x and homozygous content around 170x. **a** and **b**, show correct single haplotype mosaics with minimal level of artificial duplication. **c**, shows the separated haplotypes merged producing the duplication of content in the homozygous regions, allowing the full representation of heterozygous content. No significant artificial duplication was found (purple signal in 85x heterozygous peak and green signal in 170x homozygous peak).

**rFig. 4 (new Supplementary Figure 8). Assembly spectrum plots for evaluating *k*-mer completeness by Merqury4**. *k*-mers are colored by their presence in the ‘Rojo Pasión’ reads and OrangeRed/Currot/RojoPasión (shared) assemblies. The signal (~7%, around 85x in the black curve, representing 1-copy *k*-mers found only in the reads) corresponds to the fraction of heterozygous variants missing from the assembly plus erroneous k-mers.

**2^nd^ round**

**Reviewer 1**

Thanks to the authors for thoroughly addressing my previous comments. I know that the generation of additional data is time consuming and costly, but I think the added Hi-C data really adds to the paper in terms of both comparison and validation. Great work!
